# Neonatal supraventricular tachycardia and necrotizing enterocolitis: case report and literature review

**DOI:** 10.1186/s13052-020-00876-7

**Published:** 2020-08-26

**Authors:** Federico Mecarini, Federica Comitini, Flaminia Bardanzellu, Paola Neroni, Vassilios Fanos

**Affiliations:** Neonatal Intensive Care Unit, Department of Surgical Sciences, University of Cagliari, Cagliari, Italy

**Keywords:** Necrotizing enterocolitis, Supraventricular tachycardia, Neonatal intensive care

## Abstract

**Background:**

Necrotizing enterocolitis (NEC) and supraventricular tachycardia (SVT) are serious emergencies in the neonatal period. Although these conditions are recognized as distinct pathologies, literature reports suggest that recurrent episodes of SVT may predispose patients to NEC via disturbances in mesenteric blood flow and a decrease in tissue perfusion.

**Case presentation:**

We present a case of a preterm infant affected by recurrent episodes of SVT who developed the initial stage of NEC on the 17th day of life. Moreover, a detailed description of all the cases described in the literature is reported.

**Materials and methods:**

An integrative review of the updated literature in the Medline database and PubMed and scientific books and articles was conducted. The research from October 2019 to December 2019 was searched for with MeSH and free terms (necrotizing enterocolitis, supraventricular tachycardia) and was linked by Boolean operators.

**Conclusions:**

SVT can be considered a risk factor for the development of NEC. Therefore, clinicians should have a high level of suspicion for NEC in infants affected by SVT. This article is the first structured literature review analysing the association between SVT and NEC.

## Introduction

Necrotizing enterocolitis (NEC) is the most common gastrointestinal emergency that mainly affects premature infants in neonatal intensive care units (NICUs). NEC is considered a multifactorial disease, and its pathogenesis is not yet completely understood. Supraventricular tachycardia (SVT) is the most common pathological tachycardia in newborns, with an estimated prevalence of up to 1 in 1000 children. SVT is a heterogeneous collection of dysrhythmias characterized as a narrow-complex tachycardia originating above the level of the AV junction [1]. SVT could originate through a mechanism of retrograde conduction (reentrant tachycardia) or through atrial ectopic beats (automatic tachycardia). In the neonatal period, SVT may be asymptomatic if the episodes are brief or may present with signs of heart failure, such as poor feeding, sweating and shortness of breath, due to a prolonged episode [2]. In haemodynamically stable patients, vagal manoeuvres, such as the “diving reflex”, are the first-line nonpharmacologic therapies. If they prove ineffective, adenosine is administered. Maintenance therapy with antiarrhythmic drugs is carried out in cases of recurrent episodes, while spontaneous resolution usually occurs in the first year of life in most cases [3].

Recent data in the literature suggest a possible association between SVT and NEC. However, little is currently known about this association regarding the pathogenic mechanisms, factors that affect susceptibility, correct management and prognosis.

## Case report

We report a case of a 29^+ 6^ week preterm infant from a twin pregnancy and delivered by caesarean section. The Apgar score was 7 at 1 min and 8 at 5 min. The birth weight was 1380 g, which was appropriate for gestational age (AGA). The pregnancy was marked by twin-to-twin transfusion treated by laser therapy in utero and complicated by the death of one twin before birth. At birth, the infant was admitted to the NICU due to prematurity and respiratory distress and received ventilation support for 21 days. Parental nutrition and trophic feeding (3 ml/kg/day) were immediately started with maternal milk. The feeds gradually advanced at a rate of 10–20 ml/kg/day according to the standardized feeding regime of our NICU while monitoring for early signs of feeding intolerance. On the 13th day of life, full enteral feeding was reached (150 ml/kg/day), and parental nutrition was discontinued. Human milk fortifier (Aptamil BMF) was started at a concentration of 2.2% when the milk volume reached 100 ml/kg/day of human milk.

On the 16th day of life, the patient had recurrent episodes of SVT of 300–390 beats per minute (bpm) that were initially self-limiting or responded to vagal manoeuvres. Echocardiogram revealed a structurally normal heart with good contractility. Pharmacologic therapy with flecainide was administered orally at a dose of 4 mg/kg/day twice daily due to recurrent episodes of SVT. The infant remained haemodynamically stable with good perfusion and oxygen saturation throughout the episodes of SVT. Non-invasive blood pressure monitoring was normal, and the oxygen saturation values remained above 95% with non-invasive ventilation (high-flow nasal cannula therapy without oxygen delivery). Blood gas analysis performed after episodes of SVT did not report acid-base disorders, with a maximum lactate value of 2.1 mmol/L. Procalcitonin (PCT), C-reactive protein (CRP), blood count and urine samples were in the normal ranges. Moreover, the abdominal ultrasound assessment confirmed no evidence of hepatomegaly.

On the 17th day of life, bloody stools and abdominal distension were observed. Abdominal X-ray and ultrasound imaging were performed, showing no evidence of intestinal pneumatosis, portal venous gas or other radiographic features of NEC. The CRP levels began to increase, reaching levels of 17.8 mg/l on day 18, while PCT always remained negative. Taking into account the presence of potential NEC, enteral nutrition was discontinued, and double antibiotics (ampicillin and gentamicin) and parenteral nutrition were started. Antiarrhythmic therapy was switched to amiodarone with a maintenance dose of 5 micrograms/kg/minute, since oral flecainide was not possible. A conservative approach with bowel rest, parenteral feeding and antibiotics controlled NEC from progressing to an advanced stage, and surgical treatment was not necessary. Enteral feeding was progressively reintroduced 7 days after the onset of the initial stage of NEC without disease recurrence. He was discharged after 59 days in good clinical condition and continued antiarrhythmic therapy with 5 mg/kg/day of fleicainide administered orally in three doses.

## Discussion

A case of a preterm infant with recurrent episodes of SVT who developed the initial stage of NEC on the 17th day of life was presented. NEC is the most common gastrointestinal emergency in the NICU [4]. Although this fatal disease predominantly affects preterm infants, especially those with a very low birthweight (< 1500 g) [5], 13% of reported cases occur in full-term infants [6–8]. In these neonates, NEC mostly occurs in association with congenital heart disease (CHD) [4] that might lead to poor mesenteric artery perfusion [9–11]. NEC is often staged according to Bell’s 1978 system. Staging by Bell’s criteria has been recently supported by a statistical pattern analysis of clinical and radiological variables [12]. SVT is one of the most frequent tachyarrhythmia requiring emergency care in neonates [13]. SVT is usually asymptomatic and well tolerated in infants, but some patients may present signs of heart failure due to a prolonged episode. Although SVT management includes cardioversion using vagal manoeuvres, the primary therapy for neonates involves pharmacological agents. Our case is a preterm neonate with no structural congenital heart disease. The abdominal signs of NEC (bloody stools and abdominal distension) started after 17 days of life after full enteral feeding was achieved with maternal milk. The main identifiable risk factors for NEC in our patient were episodes of SVT, prematurity and low birth weight.

The association between NEC and SVT in newborns has been previously described in seven case reports in the literature Table 1) starting in 1998 [6, 14–17]. Two cases were of full-term infants, and four were of preterm infants. In all cases, SVT preceded the clinical onset of NEC. The first case was of a full-term infant with multiple congenital abnormalities reported by Hajivassiliou et al. in [16]. In the same year, Khalak et al. reported the first case of a preterm infant treated pre- and postnatally for SVT who developed fatal NEC 28 h after starting enteral feeding [17]. Fifteen years later, Hanna et al. reported a case of a preterm infant with recurrent episodes of SVT followed by NEC on the 11th day of life who underwent surgical resection of the compromised bowel tract [15]. Two cases of late preterm infants, one with intrauterine growth restriction (IUGR) and one large for gestational age (LGA), affected by NEC after recurrent episodes of SVT were presented by Saini et al. in 2017 [6]. The most recent case report describes a full-term infant who was breastfed and developed NEC 3 days after SVT onset [14]. The seventh case was a 15-month-old boy who presented with shock and SVT following a 12-h history of worsening abdominal pain and vomiting reported by Jacombs et al. [18]. The review focused on cases showing clinical onset in the first year of life and therefore excluded the aforementioned case.

**Table 1:** Neonatal svt and nec: case reports in literature

Case	Gestational Age	Birth Weight	Apgar score	TSV onset	Heart rate And hemodynamic parameters	NEC onset and clinical sings	Intrumental findings	Treatments	Others	References
**1**	29 + 6 Weeks	1380 g	1′: 7 5′: 8	16th day of life	- 300-390 bpm - Hemodynamically stable good perfusion	- 17th day of life - Bloody stools, abdominal distension	Abdominal X-rays: no evidence of pneumatosis	Parenteral nutrition; Antibiotics (ampicillin and gentamicin)	N.r.	clinical case of our observation and described in the text
**2**	38 Weeks	2755 g	N.r.	13th day of life	- 278-294 bpm - Echocardiography: Normal heart anatomy; borderline and dyskinetic cardiac function; Left ventricle ejection fraction of 50%	- 16th day of life - Abdominal distension	Abdominal X-rays: dilated bowel loops with no free air in the abdomen. On contrast enema severe stenosis at the transverse colon, with dilated small bowel loops	Surgery: resection of the stenotic transverse colon with end-end anastomosis, and ileal resection of a stenotic segment (2 cm) with end-end anastomosis.	Laparotomy: Terminal ileum and a jejunal loop with omentum were solidly adherent to the stenotic transverse colon	Nakib et al. 2018 [14]
**3**	36 Weeks	2100 g (IUGR)	1′: 5 5′: 6 10′: 7	3rd day of life	- 280 bpm - Hemodynamically stable. Echocardiogram: patent foramen ovale and a small patent ductus arteriosus	- 7th day of life - Rectal bleeding. Abdominal distension	Abdominal X-rays: no pneumatosis, portal venous gas or free air. Abdominal ultrasound: focal bowel wall echogenicities consistent with pneumatosis	Parenteral nutrition; Antibiotics (ampicillin, tobramycin and metronidazole)	Miller-Dieker syndrome.	Saini et al. 2017 [6]
**4**	36 Weeks	N.r. (LGA)	1′: 7 5′: 9	1st day of life	- 250 bpm - Echocardiogram: fenestrated atrial septum and restrictive patent ductus arteriosus	- 7th day of life - Bloody stools	Abdominal X-ray and abdominal ultrasound: colonic pneumatosis intestinalis	Parenteral nutrition; Antibiotics (vancomycin, tobramycin and metronidazole)	Wolff-Parkinson- White syndrome	Saini et al. 2017 [6]
**5**	35 Weeks	2200 g	1′: 5 5′: 9	Antenatal	- 240–250 bmp - Echocardiogram: structurally normal heart with good function	- 11th day of life - Emesis, abdominal distension	Abdominal X-ray: pneumatosis	Parenteral nutrition; Antibiotics Surgery: compromised bowel was resected	N.r.	Hanna et al. 2013 [15]
**6**	41 Weeks	N.r	N.r.	1 month of life	- N.r. - Echocardiogram: complex tricuspid atresia which a palliative BlalockTaussig shunt performed at 4 months of life	−5 months of life - N.r.	Abdominal X-ray: no gas consistent with ischemic injury or paralytic ileus.	Parenteral nutrition; Antibiotics Surgery: wide excision and anastomosis of ischemic small intestine	Facial and skeletal abnormalities. Laparotomy: an abscess involving the cecum and terminal part of small intestine	Hajivassiliou et al. 1998 [16]
**7**	N.r (Premature)	N.r.	N.r.	Antenatal	-N.r. - No signs of congestive heart failure	- 4th day of life - N.r.	N.r.	Fatal fulminant NEC	N.r.	Khalak et al. 1998 [17]

*LGA* Large for gestationl age, *IUGR* Intrauterine growth restriction, *NEC* Necrotizing enterocolitis, *SVT* Supraventricular tachycardia, *N.r* Not reported in the article, *Bpm* Beats per minute, *Cm* Centimeters, *g* Grams

Several theories have been formulated in an attempt to explain the origin of the correlation between SVT and NEC. The intestinal blood supply is provided by the celiac and superior and inferior mesenteric arteries, and it is widely known that ischaemia is an important contributing factor to the development of NEC [19]. A mechanism of intestinal hypoperfusion associated with NEC is the *diving reflex*, constituted by the redistribution of cardiac output to ensure brain and heart perfusion [20]. Recent literature reports suggest that recurrent episodes of SVT may predispose patients to NEC via disturbances in mesenteric blood flow [6, 14–17]. During the haemodynamic instability caused by SVT, there is an increase in vascular resistance that reduces blood flow to the mesentery, known as the “steal phenomenon”. Decreased tissue perfusion caused by diastolic backflow to areas of lower resistance [4] leads to hypoperfusion and subsequent hypoxic-ischaemic injury of the mucosa. Ischaemia induces the activation of the proinflammatory cascade and mediators such as TNF-alpha, leukotrienes, prostaglandins, complement mediators, and fragments. Furthermore, norepinephrine release and vasoconstriction seem to determine splanchnic ischaemia. The stabilization of cardiac output with control of SVT may cause oxygen free radical production with reperfusion injury. Persistent tachycardias can lead to haemodynamic compromise, but some authors suggest that NEC can also occur in infants affected by SVT without evidence of a significant haemodynamic compromise [14].

In conclusion, the exact aetiology and pathogenesis of NEC remain incompletely understood, and NEC is generally considered a multifactorial disease. Preterm birth, low birth weight, enteral feeding, infections, gastrointestinal tract immaturity and intestinal hypoxia-ischaemia are considered potential risk factors [21]. The intestinal microbiome and altered innate and adaptive host immune responses seem to play key roles. Furthermore, some authors suggest that alterations in the gut-brain axis with vagal dysregulation may also be involved in NEC pathogenesis [22].

Our case and recent reports in the literature suggest that a high level of awareness of NEC development should be maintained in the presence of SVT. Intestinal ischaemia due to SVT could have a role in causing NEC, and SVT could be a risk factor for the development of NEC. Whether SVT is simply associated with NEC or has a causative role in the pathogenesis of NEC is still under debate. Further studies are required to better identify the pathological mechanism underlying these two pathologies.

## Data Availability

Data sharing not applicable to this article.

## Abbreviations

NEC: Necrotizing enterocolitis
NICUs: Neonatal intensive care units
SVT: Supraventricular tachycardia
AGA: Appropriate for gestational age
PCT: Procalcitonin
CRP: C-reactive protein
CHD: Congenital heart disease
IUGR: Intrauterine growth restriction
LGA: Large for gestational age
